# Arboviruses: Molecular Biology, Evolution and Control. Nikos Vasilakis and Duane J. Gubler

**DOI:** 10.4269/ajtmh.16-0281

**Published:** 2016-08-03

**Authors:** Bradley Blitvich

**Affiliations:** ^1^ Iowa State University 2116 Veterinary Medicine Building Ames, IA 50011-1250 E-mail: blitvich@iastate.edu

**Figure 1:**
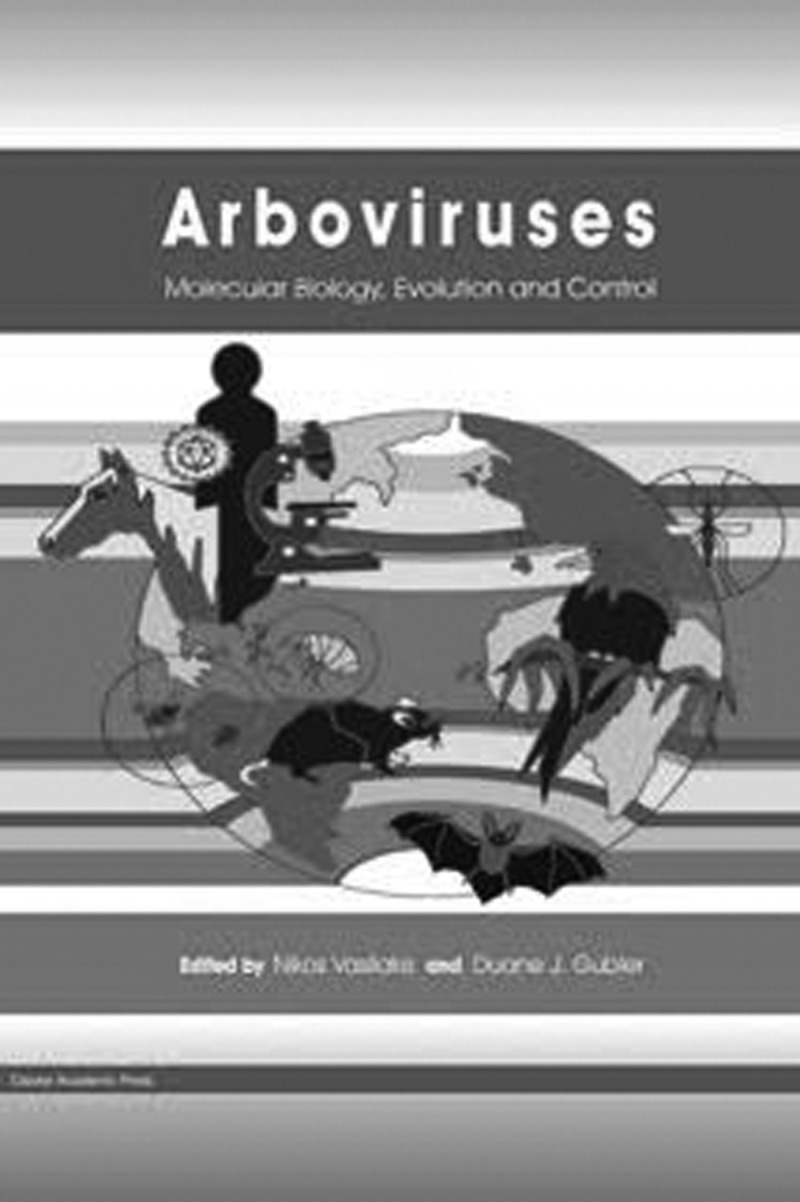

***Arboviruses: Molecular Biology, Evolution and Control.*** Nikos Vasilakis and Duane J. Gubler, eds., 2016. 398 pp. Poole, UK: Caister Academic Press. US$319, ISBN 978-1-910190-21-0

Arthropod-borne viruses (arboviruses) have a devastating impact on human health. The arbovirus of greatest medical significance is dengue virus (family *Flaviviridae*), which occurs in every, or almost every, country with a tropical or subtropical environment. According to one recent estimate, dengue virus is responsible for 390 million infections annually, of which 96 million are accompanied by clinical manifestations.^1^ Many other arboviruses are also major human pathogens; for example, yellow fever, Japanese encephalitis, West Nile, Zika, and tick-borne encephalitis viruses (*Flaviviridae*), chikungunya and Venezuelan equine encephalitis viruses (*Togaviridae*), and Crimean-Congo hemorrhagic fever, Oropouche, and severe febrile thrombocytopenia viruses (*Bunyaviridae*). Arboviruses of major veterinary significance include Rift Valley fever virus (*Bunyaviridae*) and bluetongue virus (*Reoviridae*). Most recognized arboviruses are maintained in natural transmission cycles between wild vertebrate animals and hematophagous arthropods (i.e., mosquitoes, ticks, midges, and sandflies), and cause disease after spillover transmission to humans or domestic animals that are dead-end hosts. However, some arboviruses—most notably dengue, yellow fever, chikungunya, and Zika viruses—have lost the requirement for enzootic amplification, and are maintained in human–mosquito transmission cycles. The recent emergence of Zika virus and chikungunya virus in the Western Hemisphere and the explosive disease outbreaks accompanied by these introductions provide a stark reminder of how easily arboviruses can spread across the globe and cause devastating disease in human populations. Despite the enormous burden that arboviruses impose on human and animal health, very few books devoted specifically to arboviruses have been published in recent years.

*Arboviruses: Molecular Biology, Evolution and Control*, edited by Nikos Vasilakis and Duane J. Gubler, provides a thorough and compelling review on the current status of arbovirology. The book is divided into four main sections and 22 chapters with contributions from many of the world’s leading experts in the field. The first chapter, which is preceded by an excellent foreword written by Scott C. Weaver and a preface written by the two editors, provides a concise summary on arboviruses with a particular emphasis on the taxonomic status of known, probable, and possible arboviruses (represented by eight families, 25 genera, and 492 species). The current status of arboviral disease and reasons for dramatic emergence of epidemic arboviral diseases (i.e., human population growth and unprecedented urbanization) are also discussed.

Section I (chapters 2–8) covers the molecular biology of arboviruses. Topics included in this section are taxonomy, genomic organization, replication, virus–vector and virus–host interactions, and innate immune evasion. Chapter 2 contains two appended tables; one that lists every traditional arbovirus currently recognized by the International Committee on Taxonomy of Viruses, and another comprised of viruses that infect hematophagous arthropods, but appear to have arthropod-specific host ranges. Many viruses listed in the second table were discovered within the last few years and thus, are not covered in many other virology books. The impact of next-generation sequencing technologies and other recently developed molecular approaches on virus discovery and classification is also covered. This section also compares the genomic organizations, translation products, and replication strategies of the six virus families that contain most of the arboviruses known to infect humans and livestock: alphaviruses (*Togaviridae*), flaviviruses (*Flaviviridae*), rhabdoviruses (*Rhabdoviridae*), bunyaviruses (*Bunyaviridae*), reoviruses (*Reoviridae*), and orthomyxoviruses (*Orthomyxoviridae*).

Section II (chapters 9–14) covers arboviral diversity and evolution. This section provides an excellent review of ecological and epidemiological factors that influence arbovirus genetic diversity, evolution, and emergence. The importance of host and vector genetics and virus–host interactions is also discussed. One chapter is devoted to the role of vertical transmission in the adaptation and evolution of arboviruses. The major modes of vertical transmission are explained, and the contributors include a table listing most of the bunyaviruses, flaviviruses, reoviruses, alphaviruses, rhabdoviruses, and asfaviruses known or suspected to be vertically transmitted. There is also a chapter on arbovirus genomics and metagenomics. Protocols for sample preparation, whole genome sequencing, and data analysis are provided.

Section III (chapters 15–21) covers arbovirus diagnosis and control. The opening chapter describes serologic and nucleic acid–based detection assays as well as virus isolation methods available for arbovirus diagnosis. Nucleic acid–based diagnostics is the fastest growing field in arboviral diagnosis, and assays covered in this chapter include the widely used reverse transcription polymerase chain reaction as well as less common methods such as fluorogenic assays, reverse transcription loop-mediated isothermal amplification, and nucleic acid sequence–based amplification. The proceeding chapters describe conventional methods available for arbovirus control (i.e., vector control, biological control, and vaccination), and more recent approaches that hold promise for future control efforts (i.e., the use of genetically modified vectors, RNA interference, and small molecular drug development). The chapter on vaccination summarizes the current status of dengue virus vaccine development, which has long been complicated by the potential for disease enhancement to occur after exposure to a heterologous dengue virus serotype and the unavailability of a suitable animal model. The 17D yellow fever virus vaccine, which is potentially the safest vaccine ever developed, is also discussed along with the current vaccine status of many other arboviruses of medical and veterinary significance. Section IV consists of a single chapter “Arbovirology: Back to the Future” written by Robert B. Tesh and Charles H. Calisher, two of the most recognized names in the arbovirus community. These two individuals provide the reader with a historical perspective of arbovirology and conclude with a discussion on future trends.

Overall, *Arboviruses: Molecular Biology, Evolution and Control* is an outstanding book. An impressive team of contributors was involved in its conception, and each contributor provides an excellent review of their particular research niche. This is arguably the first comprehensive book devoted specifically to arboviruses to be published in the last few decades. The book is highly recommended for every arbovirologist whether it be a first-year graduate student or an established researcher.
